# Asymmetric Functional Connectivity of the Contra- and Ipsilateral Secondary Somatosensory Cortex during Tactile Object Recognition

**DOI:** 10.3389/fnhum.2017.00662

**Published:** 2018-01-24

**Authors:** Yinghua Yu, Jiajia Yang, Yoshimichi Ejima, Hidenao Fukuyama, Jinglong Wu

**Affiliations:** ^1^Division of Medical Bioengineering, Graduate School of Natural Science and Technology, Okayama University, Okayama, Japan; ^2^Section on Functional Imaging Methods, National Institute of Mental Health, Bethesda, MD, United States; ^3^The Japan Society for the Promotion of Science, Tokyo, Japan; ^4^Beijing Institute of Technology, Beijing, China; ^5^Human Brain Research Center (HBRC), Kyoto University Graduate School of Medicine, Kyoto University, Kyoto, Japan

**Keywords:** secondary somatosensory cortex, tactile working memory, fMRI, psycho-physiological interactions, frontoparietal network

## Abstract

In the somatosensory system, it is well known that the bilateral secondary somatosensory cortex (SII) receives projections from the unilateral primary somatosensory cortex (SI), and the SII, in turn, sends feedback projections to SI. Most neuroimaging studies have clearly shown bilateral SII activation using only unilateral stimulation for both anatomical and functional connectivity across SII subregions. However, no study has unveiled differences in the functional connectivity of the contra- and ipsilateral SII network that relates to frontoparietal areas during tactile object recognition. Therefore, we used event-related functional magnetic resonance imaging (fMRI) and a delayed match-to-sample (DMS) task to investigate the contributions of bilateral SII during tactile object recognition. In the fMRI experiment, 14 healthy subjects were presented with tactile angle stimuli on their right index finger and asked to encode three sample stimuli during the encoding phase and one test stimulus during the recognition phase. Then, the subjects indicated whether the angle of test stimulus was presented during the encoding phase. The results showed that contralateral (left) SII activity was greater than ipsilateral (right) SII activity during the encoding phase, but there was no difference during the recognition phase. A subsequent psycho-physiological interaction (PPI) analysis revealed distinct connectivity from the contra- and ipsilateral SII to other regions. The left SII functionally connected to the left SI and right primary and premotor cortex, while the right SII functionally connected to the left posterior parietal cortex (PPC). Our findings suggest that in situations involving unilateral tactile object recognition, contra- and ipsilateral SII will induce an asymmetrical functional connectivity to other brain areas, which may occur by the hand contralateral effect of SII.

## Introduction

Human primary somatosensory cortex (SI) is located in the postcentral gyrus (poCG), which is the first cortical region for the perception of touch (Penfield and Boldrey, 1937; Iwamura, 1998; Bodegård et al., 2001) and contains a contralateral somatotopic organization of body representations. The secondary somatosensory cortex (SII) is a cytoarchitectonic region located bilaterally on the parietal operculum (OP), which responds to higher somatosensory processing (Eickhoff et al., 2006a,b). An early physiological study (Burton and Sinclair, 1996) indicated that compared to SI neurons, SII neurons have larger and more complex receptive field including bilateral inputs. Recently, several human neuroimaging studies (Eickhoff et al., 2008, 2010) have shown that SII is cortico-cortical connected to SI, and, therefore, SII receives and integrates tactile information from bilateral SI via the anatomical pathway. However, how SII is functionally connected to SI and whether bilateral SII play the same functional roles for unilateral somatosensory input remain poorly understood.

As stated previously, bilateral activation of SII by unilateral input is an accomplished fact; however, contralateral SII activation is markedly higher relative to the ipsilateral hemisphere (Taskin et al., 2006; Chung et al., 2014). A few recent electroencephalography (EEG) and magnetoencephalography (MEG) studies have also investigated hemispheric asymmetry of SII responses (Jung et al., 2009; Worthen et al., 2011). Specifically, Worthen et al. (2011) found significant bilateral but asymmetrical changes in neural activity that occurred in the beta-band within SI and SII using nociceptive stimulation, and Jung et al. (2009) showed asymmetry of the contralateral somatosensory evoked potential component with higher amplitudes over the contralateral side than over the ipsilateral side using median nerve stimulation. These pieces of evidence are sufficient to demonstrate hemispheric asymmetry of SII responses.

There is evidence from human functional magnetic resonance imaging (fMRI) studies (Eickhoff et al., 2006a,b, 2010; Kostopoulos et al., 2007; Chung et al., 2014) suggesting that bilateral SII serves as a higher sensorimotor node involved in complex high-level processing, ranging from somatosensory perception to sensorimotor responses. In particular, Eickhoff et al. (2010) indicated that two of the four SII subregions (i.e., OP1 and OP4) are co-activated by bilateral input, OP1 is regarded as integrating and transforming the representations that come from the SI area, and OP4 is more closely connected with motor areas for basic sensory-motor integration and/or action control. As mentioned above, Eickhoff et al. (2006a,b, 2008, 2010) studies have significantly improved the understanding of bilateral SII and their functions involved in somatosensory processing. However, these studies focused on differences in anatomical and functional connectivity across SII subregions, and no study has unveiled differences in the functional connectivity of the contra- and ipsilateral SII network that relate to frontoparietal areas.

Previous fMRI studies (Kostopoulos et al., 2007; Yang et al., 2014; Schmidt et al., 2017) using the common tactile delayed match-to-sample (DMS) paradigm always asked the subjects to feel the tactile stimulation on one hand and respond with the opposite hand. For the DMS paradigm, there is a time delay between the presentation of the sample and the matching stimuli. The subject was asked to first encode a sample stimulus and remember it. After a short delay, the subject was asked to encode the matching stimuli and make a forced-choice response to determine whether these two stimuli are matched or not during the recognition phase. Therefore, even though bilateral SII would be activated during the sample stimulus phase, it is likely that contralateral SII would be more correlated with contralateral SI than ipsilateral SII would, because contralateral SII has been shown to be recruited for sample stimulus encoding and maintenance (Kaas et al., 2013). In contrast, during the recognition phase, the SII that encodes the matching stimuli will also need to combine the sample and the matching stimuli for decision-making (Romo et al., 2002). This decision-making process is speculated to include a motor planning component for opposite hand button press. Hence, the contralateral effects of the hand will be found in SII during the recognition phase, for which the activation of contralateral SII (relative to tactile stimulation hand) is more related to somatosensory perception but the ipsilateral SII is considered to be more correlated with sensorimotor response.

In the present study, to test this hypothesis, we used an fMRI study and a tactile DMS task to investigate the functional roles of bilateral SII activation. Furthermore, we used psychophysiological interactions (PPI) analysis to estimate the functional connectivity networks of both contra- and ipsilateral SII that connect to other frontoparietal areas during the tactile DMS task, thereby revealing the functional roles of bilateral SII that are recruited in the tactile object recognition phase.

## Materials and Methods

### Subjects

Fourteen healthy right-handed male subjects (mean age 24.6 ± 0.71 years) participated in the fMRI experiment. None of the subjects reported a loss of tactile sensation; a history of major medical or neurological illness, such as epilepsy; significant head trauma; or a lifetime history of alcohol dependance. This study was carried out in accordance with the recommendations of Ethics Committee of Human and Animal Experiments, Kyoto University and Okayama University, Japan with written informed consent from all subjects. All subjects gave written informed consent in accordance with the Declaration of Helsinki. The protocol was approved by the Ethics Committee of Human and Animal Experiments, Kyoto University and Okayama University, Japan.

### Apparatus and Stimuli

**Figure 1:**
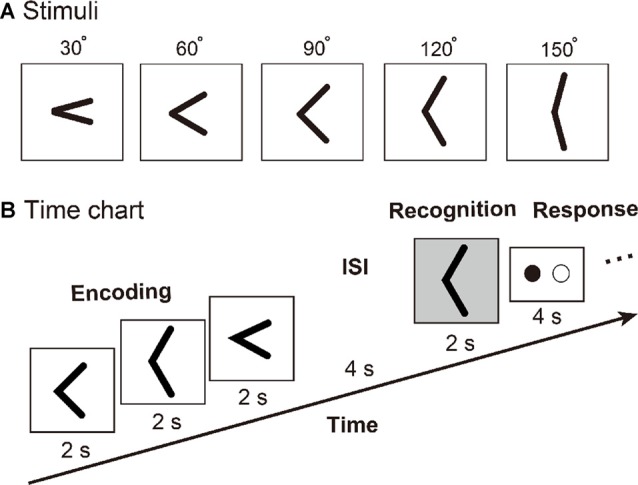
**(A)** Tactile angle stimuli. **(B)** Timing of events, for an example of the tactile angle matching task. Three angle stimuli were moved under subjects’ right index fingers during a 6-s encoding phase. Then, after a 4-s inter-stimulus interval (ISI), one angle stimulus was presented for 2 s. The subjects were asked to identify whether the last angle stimulus had been presented during the encoding phase by using the response key during a 4-s response phase.

Five custom-built plastic raised angle stimuli (from 30° to 150° proceeded by 30°) were used in this experiment (Figure 1A). These angle stimuli were raised by 0.5 mm from a 40.0-mm square base as described in our previous study (Wu et al., 2010). An MRI-compatible apparatus was used in this experiment, which can hold 16 raised angle stimuli at once, and all angle stimuli can be presented to the subject automatically by using a set of ultrasonic motors.

### Procedure

We used an event-related fMRI paradigm to assess brain activity during the tactile angle matching processing. Prior to the initiation of the fMRI experiment, all subjects were trained outside of the MR scanner until they felt comfortable performing the task. Then, the subject laid supine in the MRI tunnel with earplugs and was instructed to relax. The subject’s right arm was extended to the device and was comfortably supported by cushions. The subjects placed their right index finger lightly on the surface, with the other fingers resting on a plastic frame. The left index and middle fingers were placed on each of the two buttons of the response box. As shown in Figure 1B, three angle stimuli were moved under subject’s right index finger during a 6-s encoding phase. Then, after a 4-s inter-stimulus interval (ISI), one angle stimulus was presented for 2 s. The subjects were asked to identify whether the last angle stimulus had been presented during the encoding phase by using the response key during a 4-s response phase. The total duration of one trial was 16 s, and one of three intervals (8 s or 10 s or 18 s) followed each trial. Each subject performed a total of 72 trials, which were randomly separated into 10 runs.

### Data Acquisition

Functional MRIs were acquired on a 3T Siemens Trio whole-body MRI system. Standard sequence parameters were used to obtain the functional images as follows: gradient-echo EPI; repetition time (TR) = 2000 ms; echo time (TE) = 30 ms; flip angle = 85; 32 axial slices of 3 mm thickness with 20% slice gap; matrix = 64 mm × 64 mm; and in-plane resolution = 3.0 mm × 3.0 mm. A T1-weighted high-resolution anatomical image volume was obtained from each participant (voxel size = 1 × 1 × 1 mm^3^) before the acquisition of the functional data.

### Data Processing and Analyses

We used the Statistical Parametric Mapping (SPM12) package (Friston et al., 2007) implemented in Matlab R2007b (MathWorks, US) to process and analyze the fMRI data. Functional images from each run were realigned to the first image and then realigned to the mean image following the initial realignment. Slice-timing correction was then performed to adjust for differences in slice-acquisition times. All realigned images were co-registered to the T1-weighted anatomical image. The T1-weighted anatomical image was normalized to Montreal Neurological Institute (MNI) space using the DARTEL procedure (Ashburner, 2007). The parameters from the DARTEL procedure were then applied to each functional image as well as to the T1-weighted anatomical image. The normalized functional images were filtered using a Gaussian kernel of 8 mm full width at half-maximum (FWHM) in the x-, y- and z-axes.

After the preprocessing, a general linear model (GLM) was fit to the fMRI data for each subject (Friston et al., 1994; Worsley and Friston, 1995). The blood-oxygen-level-dependent (BOLD) signal for all tasks was modeled using box-car functions convolved with the canonical hemodynamic response function (HRF). The design matrix of each subject included 10 runs. The time series for each voxel was high-pass filtered at 1/128 Hz. Assuming a first-order autoregressive model, the serial autocorrelation was estimated from the pooled active voxels using a restricted maximum likelihood procedure and was used to whiten the data. Motion-related artifacts were minimized via the incorporation of six parameters (three displacements and three rotations) from the rigid-body realignment stage into each model. For each subject, we evaluated the linear contrasts of each task phase (encoding and recognition) relative to the baseline. We then obtained the contrast images that were used for the random-effects group analysis. To confirm bilateral SII activation during the tactile angle encoding and recognition phase, we performed a one-sample *t*-test for each contrast. The height threshold was set at *p* = 0.005 (uncorrected), and the extent threshold of activation was *p* < 0.05, which was familywise error (FWE) corrected for multiple comparisons over the whole brain. Coordinates in MNI space were labeled according to probabilistic maps (Eickhoff et al., 2005) in MNI space or the Talairach atlas after coordinate transformation into Talairach space (Lancaster et al., 2000, 2007). To confirm the activation of bilateral SII for different phases, we subsequently conducted an ROI analysis and used the SPM12 to extract the BOLD signal from the whole bilateral anatomical SII areas (Eickhoff et al., 2006a,b) of subjects.

### Psycho-physiological Interaction (PPI) Analysis

PPIs provide estimates of context-specific changes in effective connectivity between a seed region and other brain regions (Friston et al., 1997; O’Reilly et al., 2012; for review see Friston, 2011). To assess task-dependent contributions of bilateral SII to activity in other brain regions, we performed a generalized form of context-dependent PPI (gPPI) analysis (McLaren et al., 2012). We first determined the coordinates of the bilateral SII in the group analysis by evaluating mean maximum of the encoding phase and recognition phase. We then searched for the participant-specific maxima that were located within the same anatomical region (OP) and within 8 mm from the local group maximum (individual maximum). All voxels depicted by the same contrast (encoding + recognition > baseline, at a threshold of *p* < 0.05, uncorrected) within 8-mm diameter around the individual maximum served as the seed region for each subject. Time-series data were then extracted. We then calculated the PPI terms between the seed region and psychological factors in the following three steps. First, the extracted MR signal from each seed region was deconvolved with the canonical HRF. The resulting time series represented an approximation of neural activity. Second, the neural time series data were centered and multiplied by the psychological factors of (encoding phase > baseline) and (recognition phase > baseline). Finally, the interaction time series was convolved with the HRF, representing an interaction variable at the hemodynamic level (PPI term). The design matrix at the individual level included not only the PPI regressor but also the time series of the seed region, the phase effect, and regressors of no interest. We evaluated the linear contrast of the PPI regressor for each subject, and the obtained contrast image was used for subsequent group analysis (paired *t-test*). The height threshold was set at *p* = 0.005 (uncorrected), and the extent threshold of activation was *p* < 0.05, which was FWE corrected for multiple comparisons over the whole brain.

### Statistical Analyses

All statistical analyses related to the fMRI data were performed using the SPM12 package (Friston et al., 2007). The R package (R Core Team, 2014) was used for all additional statistical analyses. A one sample *t*-test was performed using the function ‘*t*.test()’ to evaluate whether the mean task accuracy exceeded the chance level. A repeated measures within-subject analysis of variance (ANOVA) was performed using the function ‘aov car()’ to evaluate the bilateral SII activation at different time points. In addition, a follow up *post hoc* test was used ‘lsmeans’() function. The significance level for all statistical tests was 0.05. The reported *p* values for the interaction, simple effect and main effect were Bonferroni-corrected.

## Results

### Behavioral Performance in the Scanner

To confirm the task performance, we calculated the mean response time (2.27 ± 0.09 s) and accuracy (64.48 ± 1.57%). We performed one sample *t*-test to compare the mean accuracy with the chance level (50%). We found that the mean accuracy significantly exceeded the chance level (*t*_(13)_ = 9.208, *p* < 0.001).

### Whole-Brain Activation during Tactile Angle Matching

Initially, we confirmed that the encoding and recognition phase of the tactile angle matching task (relative to the baseline) activated a widespread set of brain regions, including the bilateral PoCG, precentral gyrus (preCG), middle frontal gyrus (MFG), medial frontal gyrus (mFG), OP/Insula, inferior parietal lobule (IPL), intra-parietal sulcus (IPS), superior parietal lobule (SPL) and precuneus. In addition to these regions, the contrast of the recognition > baseline additionally indicated activation in the bilateral inferior frontal gyrus (IFG).

### Activation of Bilateral SII during Tactile Angle Matching

**Figure 2:**
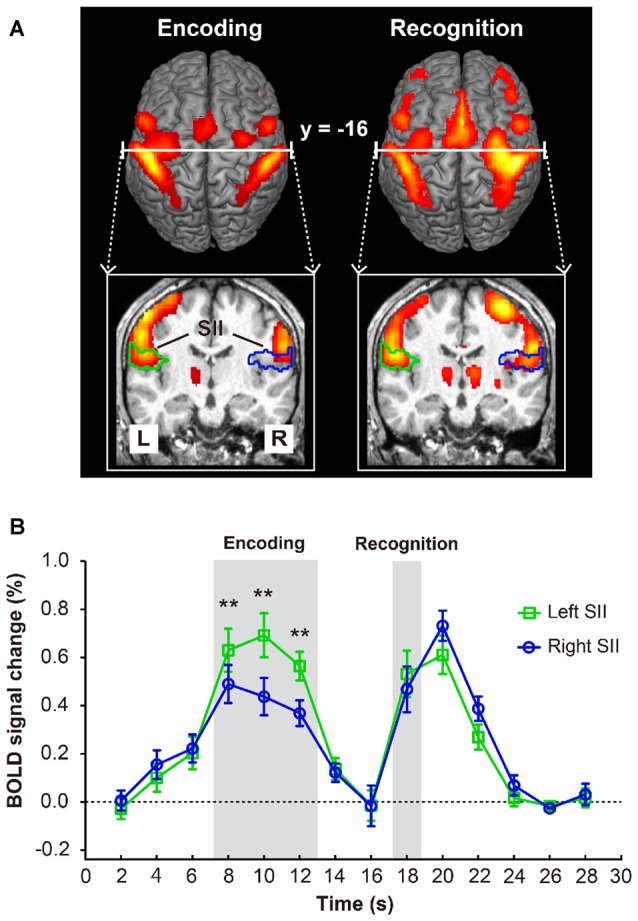
**(A)** Brain activation map of encoding and recognition phase. **(B)** The activations in bilateral secondary somatosensory cortex (SII, marked with green and blue lines) and the time course of mean blood-oxygen- level-dependent (BOLD) activity (*n* = 14) in bilateral SII. Error bars indicate ± SEM. Time points 8–12 s and 18 s (shaded gray areas) represent the BOLD activity of encoding and recognition phase. Double asterisks (**) represent the statistically significant of *p* < 0.01.

As shown in Figure 2A, both the encoding and recognition phase significantly activated bilateral SII. Then, two-way repeated measures ANOVA (2 regions × 14 levels of time points) of activity in bilateral SII revealed a significant main effect of time point (*F*_(13,169)_ = 26.667, *p* < 0.001), but we found no main effect on region (*F*_(1,13)_ = 0.657, *p* = 0.432). Moreover, we also found a significant interaction between the region and time point (*F*_(13,169)_ = 12.405, *P* < 0.001). A *post hoc* comparison revealed that the BOLD signal changes of the left SII (Figure 2B) for the encoding phase were significantly higher than those for the right SII (i.e., time points 8 [*p* = 0.0005], 10 [*p* < 0.0001] and 12 [*p* < 0.0001]); however, bilateral SII activated at the same level for the recognition phase (time point 18 [*p* = 0.101]). We also performed a repeated measures ANOVA (2 regions × 2 phases) to examine the difference between encoding and recognition phases for each region. We found a significant main effect of region (*F*_(1,13)_ = 10.286, *p* = 0.007), but we found no main effect on phase (*F*_(1,13)_ = 1.820, *p* = 0.200). In addition, we also found a significant interaction between the region and phase (*F*_(1,13)_ = 24.684, *p* < 0.001). A *post hoc* comparison revealed that the BOLD signal changes of the left SII for the encoding phase was significantly higher than that for the recognition phase (*p* = 0.039); however, right SII activated at the same level for the encoding and recognition phases (*p* = 1).

### PPI Results

**Figure 3:**
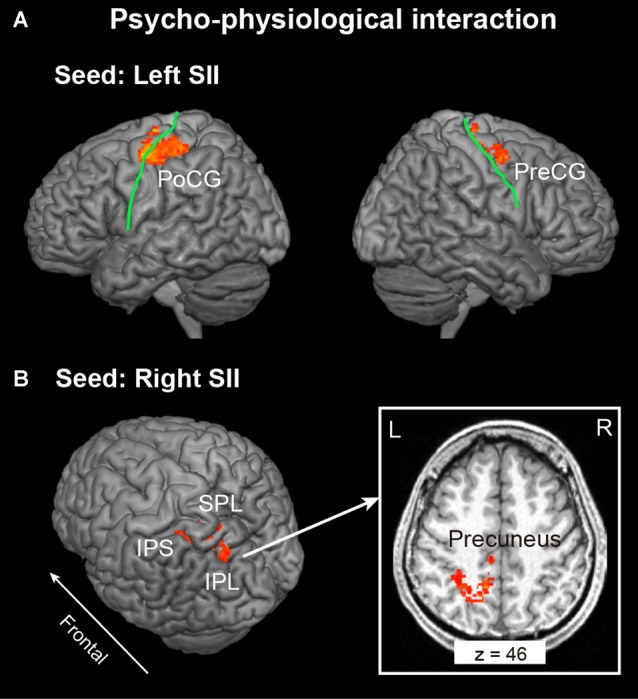
Context-dependent contributions of the **(A)** left and **(B)** right SII to brain activity in other regions were assessed using psychophysiological interactions (PPI) analysis. The extent threshold of activation was *p* < 0.05, which was familywise error (FWE) corrected for multiple comparisons over the whole brain with height threshold set at *p* < 0.005 uncorrected. The solid green line indicates the central sulcus. PreCG, Precentral Gyrus; PoCG, Postcentral Gyrus; IPS, Intraparietal Sulcus; IPL, Inferior Parietal Lobule; SPL, Superior Parietal Lobule; L, Left; R, Right.

In this analysis, bilateral SII regions were regarded as a seed region, while the task phase (recognition vs. encoding) was regarded as a psychological factor. We excluded the data of one participant from this analysis because no areas of activation met the criteria of seed selection. As shown in Figure 3A and Table 1, the PPI analysis with a left SII seed region revealed regions of significant activation in the left PoCG extended to the PreCG and right PreCG extended to the MFG. In contrast, the right SII seed PPI analysis revealed regions of significant activation in the left posterior parietal cortex (PPC), including SPL, IPL, IPS and precuneus (Figure 3B).

**Table 1:** Psycho-physiological interaction (PPI) analysis.

Spatial extent test		MNI coordinate		
Cluster size (mm^3^)	*p* value	*x*	*y*	*z*	*T* value	Hemi	Anatomical region
**PPI analysis with the left SII as a seed region**	
2848	<0.001	42	−6	52	4.58	R	PreCG
		28	−8	60	4.15	R	MFG
5272	<0.001	−32	−16	52	6.36	L	PoCG
**PPI analysis with the right SII as a seed region**	
4296	<0.001	−10	−50	46	4.91	L	Precuneus
		−30	−46	30	4.67	L	IPS
		−34	−38	32	4.08	L	IPL
		−28	−50	46	4.02	L	SPL

*The extent threshold of activation was *p* < 0.05, which was FWE corrected for multiple comparisons over the whole brain with a high threshold set at *p* < 0.005, uncorrected. PreCG, Precentral Gyrus; PoCG, Postcentral Gyrus; MFG, Middle Frontal Gyrus; IPS, Intraparietal Sulcus; IPL, Inferior Parietal Lobule; SPL, Superior Parietal Lobule; Hemi, Hemisphere; L, Left; R, Right*.

## Discussion

In the present study, we investigated the neural substrates of tactile object recognition using an event-related fMRI experiment. Our salient finding is that the functional connectivity of bilateral SII revealed different networks under recognition vs. encoding phase contrast. This finding supports our hypothesis that higher order unilateral tactile object recognition raised the asymmetrical functional connectivity of contra- and ipsilateral SII to other frontoparietal areas.

In line with previous studies (Taskin et al., 2006; Jung et al., 2009; Worthen et al., 2011; Kaas et al., 2013; Chung et al., 2014), we found that activation within left SII was greater than that of right SII during the encoding phase. Surprisingly, this functional laterality of SII was not sustained to the recognition phase (see Figure 1B), and activation of right SII was greater than that of left SII during the response phase. One straightforward interpretation of these results is that some processing that only occurred in the recognition phase may counteract the laterality. Generally, a whole DMS paradigm include information encoding, maintenance, comparison of remembered and current stimulus for decision-making. Relative to the encoding phase, the recognition phase is more specifically engaged to retrieve remembered information and make a decision. As previous non-human (Romo et al., 2002) and human (Kostopoulos et al., 2007; Eickhoff et al., 2010) studies indicated, SII has the functional role for both tactile information retrieval and decision-making; we preferred that the left-hand motor preparation process for button press of the decision-making processing was considered to counteract the laterality.

On the other hand, our PPI analysis revealed that relative to the encoding phase, the left SII is more closely integrated with areas including the left poCG, right preCG and MFG, while the right SII is more closely connected to the left SPL, IPS, IPL and precuneus. These results indicated that in situations in of unilateral tactile input, even the activation level (percent of signal change) of bilateral SII did not reveal any difference during the recognition phase; the bilateral SII regions were considered to raise the different functional network with frontoparietal areas. In the present study, the left poCG that included the left SII seed connective network was the right hand somatotopic region (Stringer et al., 2011; Ann Stringer et al., 2014). In general, the poCG is known as SI and plays an important role in touch and proprioception perception (Iwamura, 1998; Bodegård et al., 2001; Yang et al., 2017). We know from recent studies (Eickhoff et al., 2010; Yang et al., 2017) that SII is anatomically and functionally closely integrated with ipsilateral SI to receive projections from SI and in turn sends feedback projections. In the present study, this bi-directional connection between left SI and SII was considered to play an important role in retrieving encoded information from memory and compared with the current stimulus during the recognition phase. In addition, the right preCG and MFG included in the left SII seed connective network was probably the premotor cortex. This region was associated with the whole perceptual decision processing, including maintaining the first stimuli in working memory, with comparison between the two stimuli and motor commands expressing the result of the comparison (for review see Pardo-Vazquez et al., 2011). Therefore, we suggest that the psychophysiological interaction of left SII, left SI and right preCG was more correlated to the tactile angle perception and comparison process.

On the other hand, psychophysiological interaction effects were observed between the right SII and the left PPC. The left PPC is involved in sensorimotor transformations underlying planning of human actions (Andersen and Buneo, 2002) and is implicated in more cognitive functions, such as attention (Wojciulik and Kanwisher, 1999) and decision-making (Ogawa et al., 2010; Studer et al., 2015). In the present study, all subjects participated in a common tactile DMS task, and attention was considered to be kept at the same level during the task. Therefore, we preferred that the right SII and the left PPC network were likely to contribute to the whole decision-making process, including the angle comparison process, as well as the motor planning of the button-pressing action of the left hand. Planning to press a button with the left hand was expected to activate the right SII more than the left SII, which is known as contralateral effects (Ruben et al., 2001), and this effect was clearly shown during the response phase. Therefore, such contralateral effects of hand motion and motion planning might influence the brain network involved in decision-making processing.

In conclusion, the results of this study validate our hypothesis that in situations involving unilateral tactile object recognition, contra- and ipsilateral SII will induce an asymmetrical functional connectivity to the frontoparietal network. Here, we extended the findings of the previous studies not only by identifying the percent of signal change of bilateral SII but also by identifying the network implicated in the contralateral effect during tactile object recognition. The main factor result in asymmetric functional connectivity of contra- and ipsilateral SII during tactile object recognition might be the different responsibilities of the left and right hand of the DMS paradigm. In other words, left-hand tactile stimulation and the right-hand response may induce an opposite result to compare to the current finding, and this is the limitation of the present study that needs further confirmation.

## Author Contributions

JY, YY, HF and JW conceived and planned the experiments. JY and YY carried out the experiments. YY, JY, YE, HF and JW contributed to the interpretation of the results; provided critical feedback and helped shape the research, analysis and manuscript. YY took the lead in writing the manuscript.

## Conflict of Interest Statement

The authors declare that the research was conducted in the absence of any commercial or financial relationships that could be construed as a potential conflict of interest.
